# Targeting FABP4 to Inhibit AGEs‐RAGE/NF‐κB Signalling Effectively Ameliorates Nucleus Pulposus Dysfunction and Angiogenesis in Obesity‐Related Intervertebral Disc Degeneration

**DOI:** 10.1111/cpr.70021

**Published:** 2025-03-16

**Authors:** Lin Han, Fudong Li, Huiqiao Wu, Weiheng Wang, Peiwen Chen, Weicheng Xia, Yang Liu, Kaiqiang Sun, Wenbo Lin

**Affiliations:** ^1^ Department of Orthopedic Surgery, Shanghai Changzheng Hospital Naval Medical University Shanghai China; ^2^ Department of Orthopedic Surgery Third Affiliated Hospital of Naval Medical University Shanghai China

**Keywords:** angiogenesis, ECM homeostasis, FABP4, intervertebral disc degeneration, mendelian randomization, obesity, signal pathway, therapeutic target

## Abstract

Intervertebral disc degeneration (IVDD) is a primary contributor to low back pain, posing significant social and economic burdens. Increasing evidence shows that obesity contributes to IVDD, yet the underlying mechanisms remain elusive. Here, we firstly revealed a causal correlation between obesity and IVDD via a two‐sample mendelian randomization analysis and identified fatty acid‐binding protein 4 (FABP4) as the potential regulator to associate IVDD and obesity. Elevated FABP4 expression promoted extracellular matrix (ECM) disequilibrium and angiogenesis to exacerbate IVDD progression. Genetically knocking out or pharmacologically inhibiting FABP4 in high‐fat diet‐induced mice alleviated IVDD. Mechanistically, obesity activated the mammalian target of rapamycin complex 1 (mTORC1), which upregulated FABP4 expression, leading to the accumulation of advanced glycation end‐products (AGEs) in intervertebral disc tissue. AGEs further activated the NF‐κB signalling pathway, exacerbating ECM degradation and neovascularization. Conversely, rapamycin‐mediated inhibition of mTORC1 suppressed FABP4 expression in nucleus pulposus cells (NPCs), alleviating IVDD in vivo. Collectively, our findings reveal a critical role of the obesity‐induced mTORC1‐FABP4 axis in ECM degradation and angiogenesis during IVDD progression. Targeting FABP4 may represent a promising therapeutic strategy for IVDD in obese individuals.

## Graphical abstract

Obesity promotes the progression of intervertebral disc degeneration (IVDD) through the mTORC1/FABP4 axis. Targeting FABP4 effectively ameliorates nucleus pulposus dysfunction and angiogenesis in obesity‐related IVDD.

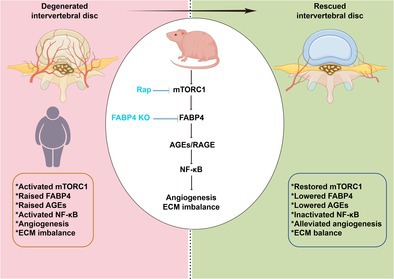

## 1 Introduction

Low back pain (LBP) is a common condition, with approximately 80% of people worldwide experiencing symptoms of LBP at some point during their lifetime [1]. Intervertebral disc degeneration (IVDD) is a major contributing factor to LBP [1]. Currently, IVDD cannot be cured, and conservative treatment is mainly used in the early stage of IVDD, including physical therapy, oral medication, lumbar muscle exercise, local nerve blockade, etc [2]. Surgical treatment is mainly used in the late stage of IVDD, but with various complications, such as cerebrospinal fluid leakage and even reoperation [2]. Gaining a deep understanding of the underlying pathological mechanisms involved in the initiation and development of IVDD will contribute to the development of novel strategies for preventing and treating this painful condition.

IVDD can be influenced by plentiful factors, of which obesity is one of the major risk factors and may accelerate the progression of degeneration [2, 3]. Numerous studies have demonstrated a strong association between obesity and IVDD, with obese individuals showing a higher prevalence and severity of disc degeneration compared to non‐obese individuals [4, 5]. Marinko et al. reported that high BMI values could increase the susceptibility of IVDD [6]. Chen et al. also found the promotive effects of obesity on IVDD progression [7]. However, the underlying mechanisms linking obesity to IVDD remain incompletely understood. Several hypotheses have been proposed to explain this relationship. One prominent theory suggests that the mechanical load imposed by excess body weight accelerates disc degeneration by increasing stress on the intervertebral discs [8]. However, this mechanical hypothesis alone cannot fully account for the observed effects, as obesity is also associated with systemic metabolic and inflammatory changes that may contribute to IVDD independently of mechanical loading [9]. Adipose tissue is not only the main energy storage site in the human body but also the largest endocrine organ, capable of synthesising and releasing a large number of bioactive adipokines that regulate energy metabolism, inflammatory responses, and other biological processes [10]. For example, elevated levels of pro‐inflammatory cytokines, such as TNF‐α and IL‐6, have been observed in obese individuals [11], which might promote disc degeneration by inducing extracellular matrix (ECM) degradation and inhibiting ECM synthesis. Additionally, dysregulated lipid metabolism in obesity leads to the accumulation of toxic lipid species, such as free fatty acids, which can induce lipotoxicity, oxidative stress, and mitochondrial dysfunction in disc cells [12]. What's more, a recent review has proposed that IVDD is in fact a kind of immunometabolism‐related disease, and abnormal adipokines might affect the pathogenesis of IVDD [2]. However, the role of adipokines in the progression of IVDD has not been fully described. Among these bioactive substances, adipocyte fatty acid‐binding protein 4 (FABP4) is a 14.6 kDa fatty acid‐binding protein highly expressed in adipocytes [13]. It can reversibly bind to saturated and unsaturated fatty acids and participate in the transport, metabolism, and storage of fatty acids [14]. Increasing evidence suggests that FABP4 is involved in processes such as inflammation and angiogenesis, which are closely associated with the pathogenesis of IVDD [15]. However, it is unclear whether FABP4 is involved in IVDD.

FABP4 has been extensively studied in the context of metabolic disorders and inflammation. In obesity, FABP4 is upregulated in adipocytes and macrophages, where it plays a critical role in lipid metabolism and insulin resistance [16]. FABP4 facilitates the intracellular transport of fatty acids and regulates their metabolic utilisation, making it a key player in lipid homeostasis [17]. Dysregulation of FABP4 has been linked to the development of metabolic syndrome and type 2 diabetes [17]. In addition to its metabolic functions, FABP4 is a potent mediator of inflammation. It promotes the secretion of pro‐inflammatory cytokines, such as TNF‐α and IL‐6, and activates inflammatory signalling pathways such as TLR4/c‐Jun and NF‐κB [18]. Given its dual role in metabolic regulation and inflammation, FABP4 represents a promising therapeutic target for diseases characterised by metabolic dysfunction and chronic inflammation. Despite these well‐documented roles of FABP4 in metabolic and inflammatory diseases, its involvement in IVDD, particularly in the context of obesity, remains poorly understood. Given that obesity is a major risk factor for IVDD and that FABP4 is a key mediator of obesity‐related metabolic and inflammatory processes, we hypothesise that FABP4 plays a critical role in obesity‐induced IVDD.

In this study, we hypothesise that FABP4 is a critical mediator in obesity‐induced IVDD, contributing to ECM disequilibrium and angiogenesis, which exacerbate IVDD progression. Our primary objectives are to investigate the causal relationship between obesity and IVDD and to identify the role of FABP4 in obesity‐induced IVDD and the molecular mechanisms by which FABP4 mediates ECM imbalance and angiogenesis. We firstly revealed the causal association between obesity and IVDD via a two‐sample Mendelian randomisation analysis based on 456,426 UKB participants of European ancestry [19]. Then, we characterised the IVD phenotype of high‐fat diet (HFD)‐induced IVDD and found that a HFD increased the level of FABP4 in IVD tissue and exacerbated the development of IVDD. Mechanically, obesity promoted the expression of FABP4 via the mTORC1 pathway, and elevated FABP4 further promoted the secretion of advanced glycation end‐products (AGEs) within IVD tissue, which resulted in activated NF‐κB signalling pathway, extracellular matrix (ECM) imbalance, and angiogenesis. Finally, pharmaceutical inhibition or knocking out FABP4 in NPCs significantly alleviated IVDD caused by HFD in vivo. Collectively, these results suggest that FABP4 could be a promising therapeutic target for IVDD.

## 2 Methods and Materials

### 2.1 Connections Between Obesity and Intervertebral Degeneration Identified Through a Two‐Sample Mendelian Randomisation Study

The genetic statistical data for intervertebral disc degeneration are derived from the FinnGen R9 release in 2023. The extensive GWAS conducted on Finnish participants includes 33,360 cases and 270,964 controls, resulting in 16,380,270 SNPs for analysis after accounting for variables like age, sex, and genotyping batches. The diagnosis of intervertebral disc degeneration is based on the International Classification of Diseases (ICD) coding standards, specifically ICD‐10 (M51), ICD‐9‐722, and ICD‐8‐725. We chose the BMI GWAS summary statistics from the GIANT consortium for BMI data, which include 2,336,260 SNPs from 681,275 participants. After excluding SNPs in linkage disequilibrium (SNP R2 > 0.001, with a physical distance of 10,000 kb), 428 SNPs were considered at the genome‐wide significance level (*p* < 5 × 10–8). The related statistical analyses were performed using the TwoSampleMR package in R (version 4.3.1), with statistical significance set at *p* < 0.05.

### 2.2 Construction of Gene Knockout Mice

The FABP4 gene knockout mice (KO) were created using the CRISPR/CAS9 system with assistance from Shanghai Model Organisms Center Inc. All animal protocols and experimental procedures were approved by the ethics board of our institution. All mice (KO and WT) were of C57BL/6J background and were held under standard 12:12 light/dark conditions in ventilated cages (≤ 5 animals/cage), a temperature of 22°C ± 2°C, and a relative humidity of 50% ± 10%, with ad libitum access to chow (Lab Diet 5053) and sterilised water. After two weeks of acclimatisation, mice were allotted to one of two dietary treatments: normal‐fat diet (NFD, 10% kcal fat; D12450Ji, Research Diets) and high‐fat diet (HFD, 60% kcal fat; D12492i). GF diets were irradiated and tested before and after experiments for sterility. The HFD mouse model was established to investigate the effects of obesity on IVDD. All animal experiments were conducted in accordance with the guidelines of the Institutional Animal Care and Use Committee (IACUC).

### 2.3 Establishment and Treatment of the Needle‐Induced IVDD Mouse Model

After two months of HDF, WT and KO mice were subjected to needle surgery to induce IVDD as previously described [20]. To ensure unbiased group allocation, mice were randomly assigned to experimental and control groups using a computer‐generated randomization scheme. The randomization was stratified by body weight to ensure balanced groups. Additionally, all surgical procedures, treatments, and outcome assessments were performed by researchers blinded to the group assignments to minimise bias. Briefly, after anaesthesia using isoflurane, the mice were placed in a prone position. Then, the appropriate coccygeal IVD segment was located and a small sagittal incision was performed. Subsequently, we used a 31G sterile needle to insert into the Co7/Co8 IVD tissue for 1.5 mm. At the same time, the needle was rotated by 180° axially and kept for 30s. Other IVD segments were left intact as the contrast. The IVD tissue would be collected at six weeks post‐surgery. For IVDD treatment experiments, the mice were administered recombinant FABP4 (HY‐P75215, MedChemExpress, 10 μM/L, 2 μL per mouse), rapamycin (HY‐10219, MedChemExpress, 10 μM/L, 2 μL per mouse), Ki8751 (HY‐12038, MedChemExpress, 10 μM/L, 2 μL per mouse), BMS309403 (HY‐101903, MedChemExpress, 10 μM/L, 2 μL per mouse), or equivalent volume of PBS into IVD tissue, respectively, twice a week for 6 weeks.

Magnetic resonance imaging (MRI) of the mouse tail was performed to assess IVDD in vivo. Mice were anaesthetised using isoflurane (4% for induction and 2% for maintenance) and placed in a prone position on a custom‐built MRI‐compatible holder. Imaging was conducted using a small animal MRI scanner equipped with a quadrature volume coil for signal transmission and reception. To ensure consistency, all MRI scans were performed by the same operator under standardised conditions. The imaging protocol was validated in experiments to confirm its sensitivity and reproducibility in detecting IVDD‐related changes in the mouse tail.

### 2.4 Histology Staining

After harvesting mouse IVD specimens, the tissues were collected and then fixed with 4% paraformaldehyde for two days, followed by decalcification in 14% ethylenediaminetetraacetic acid solution for 4 weeks, dehydrated, paraffin embedded, and sectioned at 5 μm prior to staining. The sections were stained with Haematoxylin and Eosin (HE) as well as safranin O and fast green (SOFG) to facilitate the analysis of morphological changes in the IVD tissues. To quantitatively evaluate IVDD, we employed a histological scoring system that assesses the morphology and cellularity of the annulus fibrosus (AF) and nucleus pulposus (NP), as well as the integrity of the border between these structures. The scoring system consists of five categories, with scores ranging from 0 points (normal disc) to 15 points (severely degenerated disc) [21, 22]. To ensure objectivity, histological scoring was performed by two independent researchers who were blinded to the group assignments.

### 2.5 Immunohistochemical (IHC) Staining

The sections were initially deparaffinised using xylene and then rehydrated with ethanol for IHC staining. For antigen retrieval, a citrate buffer solution with a 0.1 mol·L^−1^ concentration and a pH of 6.0 was used. After blocking with a peroxidase‐blocking solution and normal horse serum, the sections were incubated with primary antibodies at 4°C overnight. Next, the sections were exposed to biotinylated IgG and streptavidin‐horseradish peroxidase, and the immunoreactivity was visualised with the DAB Peroxidase Substrate Kit (G1212, ServiceBio). Finally, the sections were counterstained by haematoxylin and then mounted. The antibodies used for IHC staining included Aggrecan (ACAN, DF7561, Affinity), MMP3(340,612, ZenBio), and COL2A1 (AF0135, Affinity). All IHC staining and analyses were performed by researchers blinded to the experimental groups.

### 2.6 Immunofluorescence (IF) Staining

For IF staining, the sections were prepared similarly to those for IHC staining. After being blocked with IF staining blocking Buffer (G2010, ServiceBio) containing Triton 100 (G1204, ServiceBio), the sections were incubated with primary antibodies (4°C, overnight). Subsequently, the sections were treated with secondary antibodies tagged with either anti‐mouse/rabbit Alexa Fluor 488 or 568. Finally, the sections were examined under a confocal microscope and analysed with ImageJ software. The antibodies used for IF staining included FABP4 (abcam, ab92501), MMP3(340,612, ZenBio), COL2A1 (AF0135, Affinity), Emcn (abcam, ab106100), CD31 (abcam, ab7388), p65 (ab131100, abcam), p‐p65 (GB113882, ServiceBio), RAGE (ab228861, abcam), and PS6 (GB115635, ServiceBio). IF staining and quantification were performed by researchers blinded to the group assignments.

### 2.7 Isolation and Culture of Human Primary NPCs

This method has been described previously [21]. Before the study, the informed consent was obtained from patients. In brief, during surgery, the NP tissues were transported to an ultra‐clean lab in a 0.9% sodium chloride solution. They were then washed three times with sterilised PBS (G0002, Servicebio) before being treated with 0.25% Trypsin–EDTA (Servicebio, catalogue number G4001) for 30 min, and subsequently with 0.2% collagenase type II (Invitrogen) for an additional hour under shaking conditions at 37°C and 75 rpm. Finally, The separated cells were mixed into a complete DMEM/F‐12 medium that included 10% fetal bovine serum and 1% penicillin–streptomycin, and then cultivated in a T25 culture flask under aseptic conditions with 5% CO_2_ at 37°C. A concentration of 200 μM palmitic acid (PA) was used to treat NPCs to induce lipid accumulation in NPCs [23]. The concentration of PA was selected based on a previous study, ensuring that it induced significant lipotoxic effects without causing excessive cell death, allowing us to study the mechanisms underlying obesity‐induced IVDD in a physiologically relevant context.

### 2.8 Quantitative Real‐Time Polymerase Chain Reaction (qRT‐PCR)

Total RNA was extracted from both NP tissues and human NPCs using Trizol reagent (Invitrogen, Carlsbad, CA, USA), followed by reverse transcription and amplification using HiScript III RT SuperMix for qPCR Kit (R323‐01, Vazyme) on a Real‐Time PCR system (Applied Biosystems, Foster City, USA) [21]. Then, the relative expression of genes was visualised using SYBR qPCR Master Mix (Q711‐02, Vazyme). The genes' relative expression was determined using the formula: 2^−ΔΔCt^. The primers used for qRT‐PCR were as follows:

Human, FABP4, forward 5′‐AGATTTCCTTCATACTGGGC‐3′, reverse 5′‐AAGGTTATGGTGCTCTTGACT‐3′.

Human, FABP1, forward 5′‐AGCACTTCAAGTTCACCATCA‐3′, reverse 5′‐TTTCTCCCCTGTCATTGTCTC‐3′

Human, FABP2, forward 5′‐CCATGTTGCTTTATATGTAGCC‐3′, reverse 5′‐CTACATTCCAGCCTGAGTGA‐3′.

Human, FABP3, forward 5′‐CACCACATTGCCTCATTTCTT‐3′, reverse 5′‐CATGGGAACTGGAACTGGAT‐3′.

Human, FABP5, forward 5′‐GTCACCTGTACTCGGATCTATGA‐3′, reverse 5′‐GAAAGAAACAGTATGGAGATTTGC‐3′.

Human, FABP6, forward 5′‐GGGATCTCCAGCGATGTAAT‐3′, reverse 5′‐TAGTGCTGGGACCAAGTGAA‐3′.

Human, FABP7, forward 5′‐GGAAATGTGACCAAACCAA‐3′, reverse 5′‐GCTGGAAACTAATCTCCGTG‐3′.

Human, FABP12, forward 5′‐TGGCAAAACCCACTGTGACCAT‐3′, reverse 5′‐CCTGGCGTGATTTCCTCAAACTC‐3′.

Human, Col2α1, forward 5′‐TGGACGATCAGGCGAAACC‐3′, reverse 5′‐GCTGCGGATGCTCTCAATCT‐3′.

Human, Aggrecan, forward 5′‐ACTCTGGGTTTTCGTGACTCT‐3′, reverse 5′‐ACACTCAGCGAGTTGTCATGG‐3′.

Human, MMP3, forward 5′‐CTGGACTCCGACACTCTGGA‐3′, reverse 5′‐CAGGAAAGGTTCTGAAGTGACC‐3′.

Human, MMP13, forward 5′‐ATCTGAACTGGGTCTTCCAA‐3′, reverse 5′‐GCCTGTATCCCTCAAAGTGAAC‐3′.

Human, GAPDH, forward 5′‐TCAAGAAGGTGGTGAAGCAGG‐3′, reverse 5′‐TCAAAGGTGGAGGAGTGGGT‐3′.

Mouse, FABP4, forward 5′‐CAC CGA GAT TTC CTT CAA ACT‐3′, reverse 5′‐GTT ATG ATG CTC TTC ACC TTC C‐3′.

GAPDH was used for normalisation. The experiments were performed in triplicate.

### 2.9 Bulk RNA Sequencing and Bioinformatics Analysis

For in vitro cell experiments, after culture of human NPCs for 24 h, rmFABP4 (HY‐P75215, MedChemExpress, 10 ng/mL) was added onto the culture medium for another 24 h. For in vivo experiment, after two months of HDF, WT and KO mice were subject to needle surgery to induce IVDD, after the two weeks of the surgery, the IVD tissue was collected. After preparation of human NPCs and IVD tissue from mice models, total RNA was isolated according to the instructions [21]. Later, Genekinder Medicaltech (Genekinder Medicaltech Co. Ltd., China) conducted mRNA‐seq and analysed NPCs or mouse IVD tissue. Total RNA was extracted using the TRIzol reagent according to the manufacturer's instructions. Samples RNA integrity numbers (RIN) > 8.0 were considered acceptable for sequencing. Quality control of raw sequencing data was performed to assess read quality, GC content, and potential adapter contamination. Low‐quality reads (Phred score < 20) and adapter sequences were trimmed using Trimmomatic (version 0.39). Gene expression levels were quantified and normalised to transcripts per million (TPM) for downstream analysis. Differential gene expression analysis was performed using DESeq2 (version 1.30.1) in R (version 4.0.3). The R package Deseq2 was employed for analysing differentially expressed genes (DEGs). These DEGs were then visualised using volcano and heatmap plots. Functional enrichment analysis of differentially expressed genes was performed using the Gene Ontology (GO) and Kyoto Encyclopedia of Genes and Genomes (KEGG) databases.

### 2.10 Enzyme‐Linked Immunosorbent Assay (ELISA)

Serum samples or cell supernatant were isolated from mouse blood or human NPCs at the terminal end of the experiment. FABP4 level (E‐EL‐M2404, Elabscience) and VEGF level (E‐EL‐H0111, Elabscience) were measured following the instructions of the ELISA kit.

### 2.11 The Human Umbilical Vein Endothelial Cells (HUVECs) Scratch‐Wound Assay

HUVECs were purchased from Pricella (Wuhan, China) and cultured in endothelial growth medium (Cat#CM‐0122, Pricella, Wuhan, China) according to the manufacturer's instructions. Cells were used between passages 3 and 6 to ensure consistency in experimental conditions. HUVECs were placed in 6‐well culture plates and left to reach confluence in α‐MEM containing 10% FBS for 24 h. Afterwards, a scratch was made using a 200‐μl yellow plastic pipette tip to create a cell‐free gap, which served as a measure of cell migration. The cultures were rinsed with PBS to eliminate cells that did not adhere. Afterward, the HUVEC cultures were kept in α‐MEM medium without FBS. Images of the scratch were taken at set intervals with an inverted phase‐contrast microscope (Nikon Instruments Inc., Melville, NY, USA) equipped with a CCD camera. Images were analysed using the ImageJ software (National Institutes of Health, Bethesda, MD, USA).

### 2.12 The HUVECs Tube‐Formation Assay

HUVECS were placed into 12‐well plates that had been pre‐coated with Matrigel Matrix Growth Factor Reduced (BD Biosciences, Franklin Lakes, NJ, USA) at a density of 3 × 10^3^ cells/well and cultured at 37°C for 6 h. The cells underwent a one‐hour phalloidin staining process, followed by counting the branches. A fluorescence microscope (Olympus, Tokyo, Japan) was employed to count tube branches at ×10 magnification, measure tube length in pixels, and tally the capillary network meshes, nodes, and branches across five random fields per culture plate well.

### 2.13 Statistical Analysis

The Prism software package (GraphPad 9.0 6, USA) was used to conduct statistical analyses. Differences between groups were statistically evaluated using either the Student's t‐test or ANOVA, with Tukey's post hoc test applied afterward. For ordinal data, the Kruskal‐Wallis test was applied, followed by Dunn's post hoc test. Unless stated differently, most of the data shown represent at least three separate experiments and are presented as mean ± SEM. Statistical significance was defined as a *p*‐value less than 0.05 (* indicates *p* < 0.05, ** indicates *p* < 0.01, *** indicates *p* < 0.001, and **** indicates *p* < 0.0001).

## 3 Results

### 3.1 Obesity Promoted the Progression of IVDD

**FIGURE 1:**
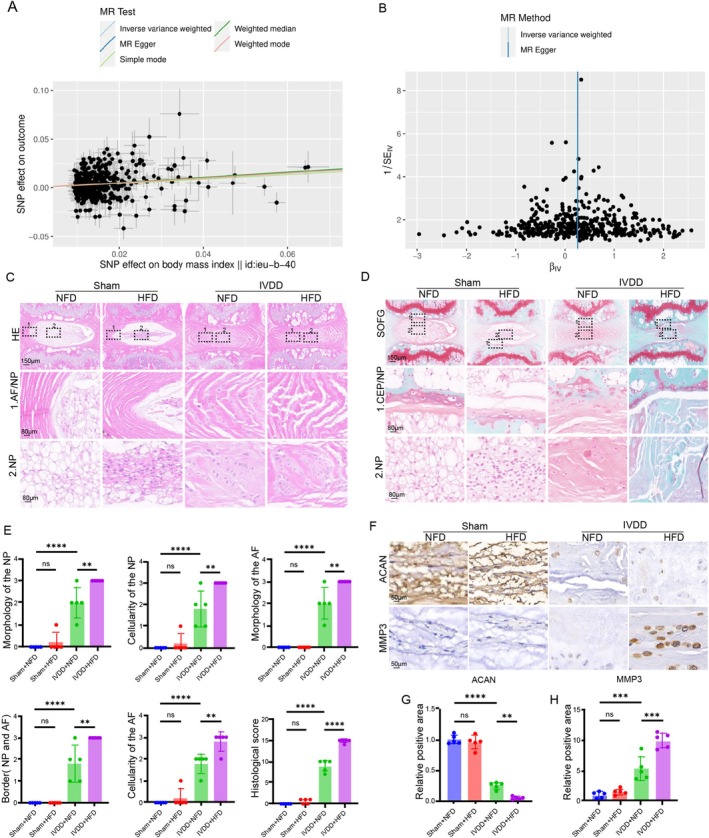
Obesity increased the risk of IVDD (A) The impact of genetically correlated BMI and obesity‐related traits on IVDD was assessed using scatter plot calculations employing methods such as Inverse Variance Weighting (IVW), MR‐Egger, Simple Mode, Weighted Median, and Weighted Mode. (B) The overall symmetry of the funnel plot indicates that there is no publication bias or other systematic bias in the MR study results. (C) H&E staining of the IVD tissue from mice in different groups. (D) SOFG staining of the IVD tissue from mice in different groups. (E) Histological score of the IVD tissue from mice in different groups, including morphology, cellularity, and the bonder between NP and AF tissue (*n* = 5). (F) IHC staining for ACAN and MMP3 the IVD tissue from mice in different groups. (G and H) Quantitation results of the IHC staining for ACAN and MMP3 the IVD tissue from mice in different groups (*n* = 5). *p < 0.05, **p < 0.01, ***p < 0.001, and ****p < 0.0001.

Previous investigations have highlighted a potential association between obesity and IVDD [2, 3, 24]. To deeply investigate the relationship between obesity and IVDD, we firstly performed a two‐sample mendelian randomization analysis. We used univariate Mendelian randomization (MR) to assess the effect of BMI on intervertebral disc degeneration. Across various methodological models, BMI was generally significantly associated with IVDD. Specifically, the odds ratio (OR) using the inverse variance weighted (IVW) method was 1.28 (95% CI, 1.19–1.37, *p* = 2.42 × 10–12), the OR using the MR Egger method was 1.20 (95% CI, 1.07–1.54, *p* = 0.006), the OR using the weighted median method was 1.31 (95% CI, 1.19–1.44, p = 2.42 × 10–8), the OR using the simple mode method was 1.25 (95% CI, 0.92–1.69, *p* = 0.14), and the OR using the weighted mode method was 1.28 (95% CI, 1.08–1.53, *p* = 0.003). No significant pleiotropy was found in all analyses (Figure 1A,B). In conclusion, the MR study provided evidence that obesity was associated with the risk of IVDD.

Then, we firstly used HFD to induce obesity in mice for 3 months and then established the needle‐induced IVDD model to explore the IVD phenotype alterations in the obesity state. As shown in the results of H&E staining, after being fed with an HFD for 3 months, the area of NP tissue experienced a slight decrease, although there was no statistically significant difference (Figure 1C,E). However, in terms of the IVDD group, HFD evidently accelerated the progression and severity of IVDD (Figure 1C,E). H&E staining showed that compared with the NFD + IVDD group, the NP tissue was nearly disappearing and the boundary between NP and AF tissue was not clear in the HFD + IVDD group (Figure 1C,E). We further evaluated the proteoglycan content within the IVD tissue using Safranine O‐Fast Green (SOFG) staining, and the results showed that many NPCs with large vacuoles and more proteoglycan content existed in the gelatinous structure of NP tissues from the sham group, whereas needle surgery markedly resulted in the disappearing vacuoles and reduced proteoglycans in NPCs in the two IVDD groups (Figure 1D,E). However, mice in the HFD + IVDD group showed a more severe decrease in the proteoglycans content (Figure 1D,E). NPCs are important contributors to ECM synthesis and metabolism, maintaining the gel‐like nature of the NP tissue to enable it to withstand various mechanical stresses. The reduction in the number of NPCs and the imbalance of ECM synthesis and degradation are important factors for IVDD [2]. We further evaluated the expression of the ECM homeostasis‐related markers, ACAN and MMP3, in the IVD tissue, and the IHC staining suggested that needle surgery significantly decreased the expression of ACAN and increased the expression of MMP3. Nevertheless, the administration of HFD resulted in more severe alterations of ECM imbalance (Figure 1F–H).

These findings indicated that a high‐fat diet might worsen the degenerative changes in intervertebral disc tissue, implying that obesity is a significant risk factor for the advancement of intervertebral disc degeneration.

### 3.2 Identification of FABP4 as the Critical Regulator for Obesity‐Induced IVDD

**FIGURE 2:**
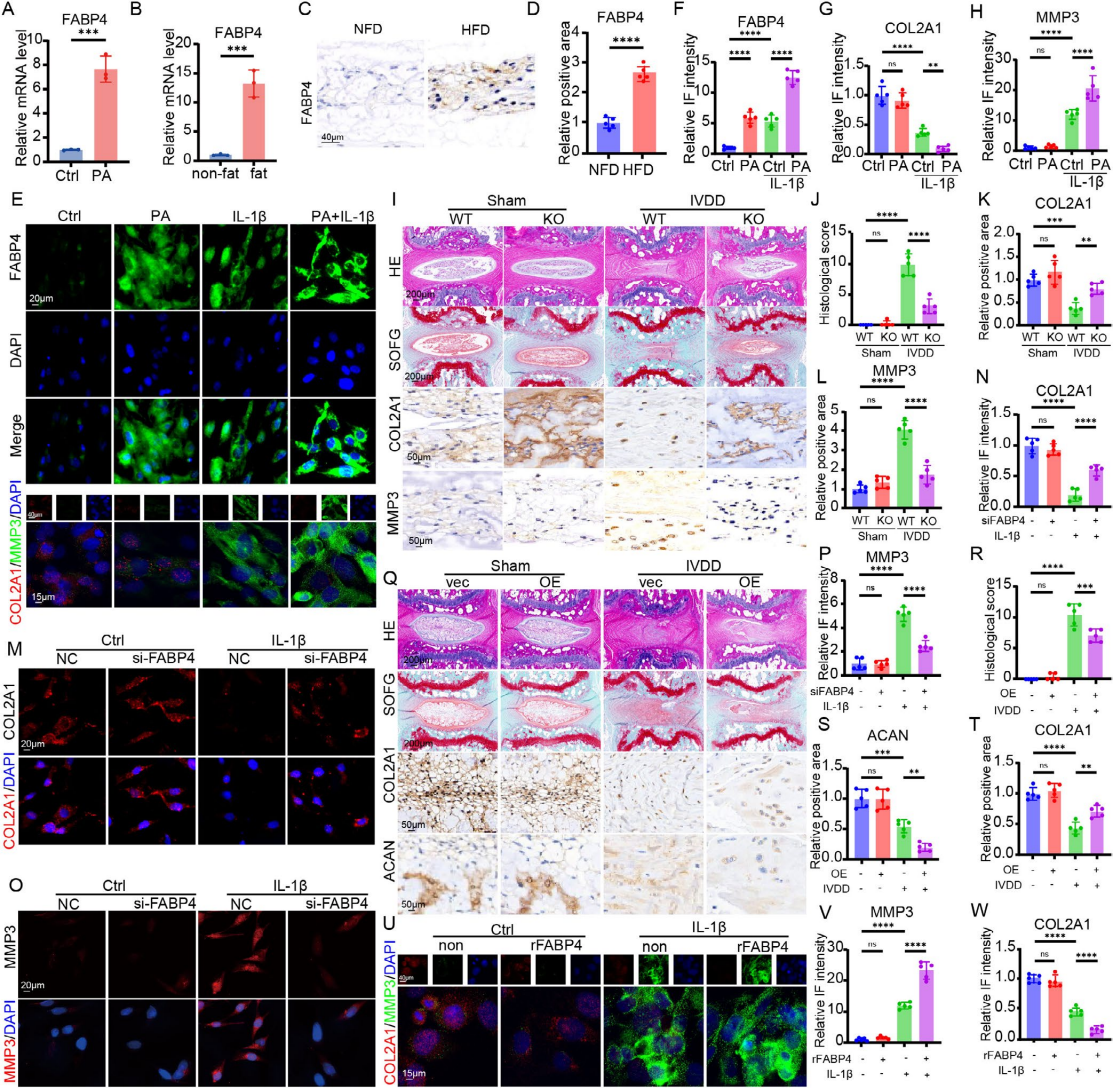
FABP4 played a critical role in mediating obesity‐related IVDD. (A) RT‐qPCR analysis of the expression of FABP4 in NPCs under lipotoxic condition (*n* = 3). (B) RT‐qPCR analysis of the expression of FABP4 in IVD tissue from obesity and none‐obesity mice (*n* = 3). (C) IHC staining for FABP4 in IVD tissue from obesity and none‐obesity mice. (D) Quantitation result of IHC staining for FABP4 in IVD tissue from obesity and none‐obesity mice (*n* = 5). (E) IF staining for the expression of FABP4, COL2A1, and MMP3 in NPCs under lipotoxic condition with or without IL‐1β. (F–H) Quantitation results of the IF staining for the expression of FABP4, COL2A1, and MMP3 in NPCs under lipotoxic condition with or without IL‐1β (*n* = 5). (I) Degeneration evaluated by H&E, SOFG, and IHC staining of IVD tissue in WT and FABP4‐KO mice from sham and IVDD group, respectively. (J) Histological score of IVD tissue in WT and FABP4‐KO mice from sham and IVDD group, respectively (*n* = 5). (K and L) Quantitation results of IHC staining for ACAN and MMP3 of the IVD tissue in WT and FABP4‐KO mice from sham and IVDD group, respectively (*n* = 5). (M) IF staining for COL2A1 in IL‐1β‐induced NPCs with or without silencing FABP4. (N) Quantitation result of IF staining for COL2A1 in IL‐1β‐induced NPCs with or without silencing FABP4 (*n* = 5). (O) IF staining for MMP3 in IL‐1β‐induced NPCs with or without silencing FABP4. (P) Quantitation result of IF staining for MMP3 in IL‐1β‐induced NPCs with or without silencing FABP4 (*n* = 5). (Q) Degeneration evaluated by H&E, SOFG, and IHC staining of IVD tissue in blank and FABP4‐overexpressed mice from sham and IVDD group, respectively. (R) Histological score of IVD tissue in blank and FABP4‐overexpressed mice from sham and IVDD group, respectively (*n* = 5). (S and T) Quantitation results of IHC staining for ACAN and MMP3 of the IVD tissue in blank and FABP4‐overexpressed mice from sham and IVDD group, respectively (*n* = 5). (U) IF staining for COL2A1 and MMP3 in IL‐1β‐induced NPCs with or without the administration of recombinant FABP4. (V and W) Quantitation results of the IF staining for COL2A1 and MMP3 in IL‐1β‐induced NPCs with or without the administration of recombinant FABP4 (*n* = 5). *p < 0.05, **p < 0.01, ***p < 0.001, and ****p < 0.0001.

FABP family proteins play an important role in the development of obesity and high‐fat‐related diseases, especially in chronic low‐grade inflammation [15]. To mimic the lipotoxic environment associated with obesity, we established a palmitic acid (PA)‐treated NPC lipotoxic model [25]. PA is the most abundant saturated free fatty acid in the human body and is significantly elevated in the serum of obese individuals [26]. Elevated PA levels are known to induce lipotoxicity, inflammation, and metabolic dysfunction, which are key features of obesity‐related pathologies [27]. Therefore, the PA‐treated NPC model serves as an in vitro system to study the effects of obesity‐induced lipotoxicity on IVDD. The mRNA expression of FABPs (1‐7 and 12) in NPCs was tested. The results of RT‐qPCR showed that, among those FABPs, the increase in FABP4 expression was the most obvious (Figures 2A and S1A). To further explore the regulatory mechanism of FABP4 during obesity‐induced IVDD, we firstly evaluated the expression of FABP4 in mouse IVD tissue. The results of RT‐qPCR suggested that the expression of FABP4 increased significantly in HFD‐induced IVD tissue compared to NFD‐induced IVD tissue (Figure 2B). Notably, the serum level of FABP4 was also higher in the HFD group than in the NFD group (Figure S1B). IHC staining showed that the protein expression of FABP4 was also elevated in HFD‐induced IVDD tissue (Figure 2C,D). In addition, we introduced IL‐1β to mimic the needle injury and examined the expression of FABP4 at the cellular level. The results of IF staining suggested that the administration of PA or IL‐1β contributed to an evident increase in the protein expression of FABP4 and that the combination of PA and IL‐1β further enhanced the expression of FABP4 (Figure 2E,F). Notably, the combination of PA and IL‐1β also resulted in a dramatic alteration in the expression of COL2A1 and MMP3 (Figure 2E,G,H). The results above indicated that FABP4 can be induced both in obesity and in an inflammatory state.

Subsequently, we constructed a FABP4‐knockout mice model to investigate the impact of FABP4 on IVDD in an obese state. Then, we established an HFD mice model with or without needle surgery. At the terminal point of the experiment, we collected the IVD tissue to evaluate the degeneration state. H&E staining and SOFG staining showed that in the state of HFD, the volume and proteoglycan content of NP tissue in the FABP4‐KO sham group showed no significant statistical difference compared to that in the WT sham group (Figure 2I,J). However, in the IVDD group, mice from the FABP4‐KO group manifested a relatively larger volume of NP tissue, higher proteoglycan content, and a relatively clearer boundary between NP and AF tissue (Figure 2I,J). In addition, we also detected the alterations of ECM metabolism and found that FABP4 deficiency significantly alleviated the disequilibrium of ECM in terms of the expression of COL2A1 and MMP3 in the obese state (Figure 2I,K,L). In vitro experiments also showed that silencing FABP4 could alleviate inflammation‐induced ECM degradation within NPCs (Figure 2M–P). To further test the relationship between FABP4 and obesity‐induced IVDD, we established a FABP4‐overexpression mice model using lentivirus. After intradiscal administration of lenti‐FABP4 (OE) or lenti‐NC (Vector) for 7 days, the HFD model was established for 3 months, followed by needle surgery. After one month of IVDD surgery, we collected the IVD tissue and evaluated the histological changes of the IVD tissue. As shown in the results, in the Vector group, the needle resulted in severe degenerated changes of the IVD, including decreased volume of NP tissue and the boundary between NP and AF tissue indicated by H&E and SOFG staining (Figure 2Q,R). Nevertheless, overexpression of FABP4 contributed to more disturbed tissue structure of the IVD, including nearly disappearing NP tissue and disorganised AF tissue (Figure 2Q,R). The results of IHC staining also showed that overexpression of FABP4 further exacerbated obesity‐mediated IVDD (Figure 2Q,S,T).

We also evaluated the bio‐effects of FABP4 on IL‐1β‐treated NPC model in vitro. The results showed that the presence of IL‐1β decreased the expression of COL2A1 and increased the expression of MMP3; in addition, recombinant FABP4 (rFABP4) dramatically aggravated this effect (Figure 2U–W).

Collectively, these experiments above demonstrated that FABP4 was the critical regulator for obesity‐induced IVDD, and inhibiting the expression of FABP4 showed favorable effects on obesity‐related IVDD.

### 3.3 FABP4 Contributed to Dysfunction and ECM Unbalance in NPCs via Activating AGEs/RAGE/NFκB Signalling Pathway

**FIGURE 3:**
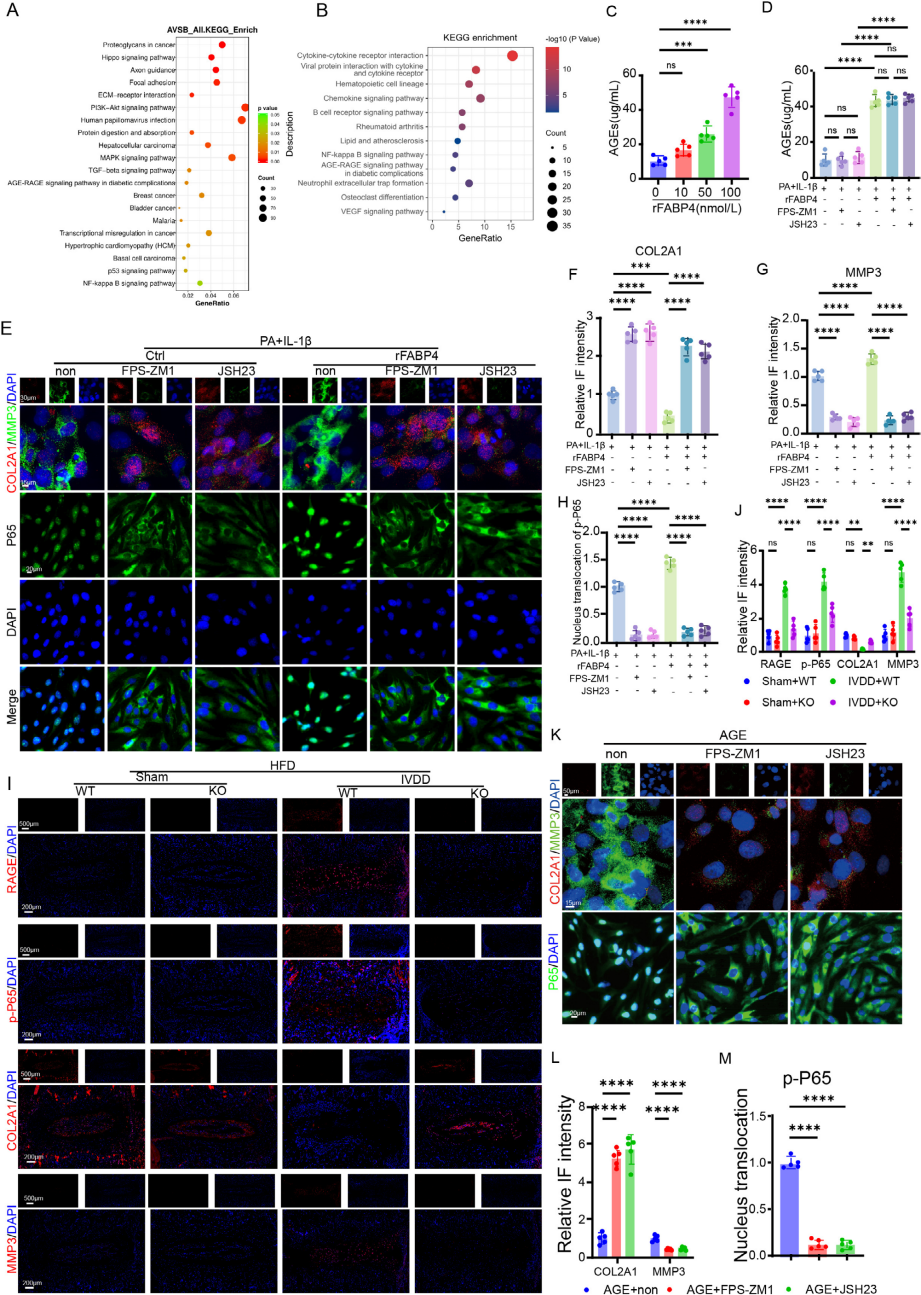
FABP4 contributed to dysfunction and ECM unbalance in NPCs via activating AGEs/RAGE/NFκB signalling pathway. (A) KEGG analysis of the NPCs with or without the administration of rFABP4 (*n* = 3). (B) KEGG analysis of the IVD tissue from WT and KO mice (*n* = 5). (C) ELISA result of AGEs level in the supernatant of the NPCs treated by rFABP4 in a dose‐dependent manner (*n* = 5). (D) ELISA result of AGEs level in the supernatant of the NPCs under lipotoxic condition with or without the treatment of rFABP4, FPS‐ZM1 (an inhibitor for RAGE), and JSH23 (an inhibitor for p65) (*n* = 5). (E) IF staining for COL2A1, MMP3, and p65 in NPCs under lipotoxic condition with or without the treatment of rFABP4, FPS‐ZM1, and JSH23. (F–H) Quantitation results of the IF staining for COL2A1, MMP3, and p65 in NPCs under lipotoxic condition with or without the treatment of rFABP4, FPS‐ZM1, and JSH23 (*n* = 5). (I) IF staining for RAGE, p‐p65, COL2A1, and MMP3 in WT and KO mice from sham or IVDD groups under HFD condition, respectively. (J) Quantitation results of the IF staining for RAGE, p‐p65, COL2A1, and MMP3 in WT and KO mice from sham or IVDD groups under HFD condition, respectively (*n* = 5). (K) IF staining for COL2A1, MMP3, and p65 in NPCs treated by AGEs with or without the treatment of FPS‐ZM1 and JSH23. (L and M) Quantitation results of the F staining for COL2A1, MMP3, and p65 in NPCs treated by AGEs with or without the treatment of FPS‐ZM1 and JSH23 (*n* = 5). *p < 0.05, **p < 0.01, ***p < 0.001, and ****p < 0.0001.

To further investigate the intrinsic mechanism of FABP4 involved in IVDD, we collected the IVD tissue from either HFD‐induced or NFD‐induced mice, followed by bulk RNA sequencing. In addition, we also treated human NPCs with recombinant FABP4 and performed the bulk RNA sequencing. Analysis of KEGG in vivo and in vitro suggested that either obesity or administration with rFABP4 activated both AGEs/RAGE and NFκB signalling pathways (Figure 3A,B). Firstly, we evaluated the AGEs level in NPCs induced with rFABP4 and found that rFABP4 could dose‐dependently increase the secretion of AGEs (Figure 3C). In addition, to explore whether the promotive effect of rFABP4 on the production of AGEs was mediated by the AGEs/RAGE or NFκB signalling pathway, we treated rFABP4‐induced NPCs using either FPS‐ZM1 (an inhibitor for RAGE) or JSH23 (an inhibitor for NFκB signalling pathway). The results showed that rFABP4 increased the production of AGEs, whereas this effect was not affected by JSH23, which may suggest that rFABP4 showed a regulative effect on AGEs/RAGE and that AGEs/RAGE could not be regulated by NFκB signalling pathway (Figure 3D). To test this hypothesis, we performed the in vitro experiments using an obesity‐induced IVDD cell model. IF staining showed that in the absence of rFABP4, the administration of either FPS‐ZM1 or JSH23 significantly decreased the expression of MMP3 and increased the expression of COL2A1 (Figure 3E–G). Importantly, after the administration of rFABP4, FPS‐ZM1 and JSH23 also showed a significant therapeutic effect on ECM homeostasis, including decreased expression of MMP3 and increased expression of COL2A1 (Figure 3E–G). The above results demonstrated that both AGEs/RAGE and NFκB signalling pathways played an important role in modulating FABP4‐associated ECM degradation. Notably, IF staining for p65 indicated that blocking AGEs/RAGE showed a similar inhibitory effect on the nuclear translocation of p65 to JSH23 (Figure 3E,H), indicating that NFκB signalling pathway was regulated by the AGEs/RAGE pathway under the condition of FABP4 treatment.

We also examined the activation of AGEs/RAGE and NFκB signalling pathway in obesity mouse models. As the results indicated, in the presence of HFD, knocking out FABP4 significantly decreased the ratio of RAGE‐ and p‐p65‐positive NPCs within NP tissue (Figure 3I,J). Furthermore, inhibiting the activation of NFκB signalling pathway also alleviated AGEs‐induced NPC dysfunction, and the therapeutic effect was similar to that of direct blocking of AGEs/RAGE signalling (Figure 3K–M).

Taken together, FABP4 mediated obesity‐related IVDD via dysfunction mainly by activating AGEs/RAGE/NFκB signalling cascade.

### 3.4 Obesity‐Induced Expression of FABP4 Depended on the mTORC1 Pathway

**FIGURE 4:**
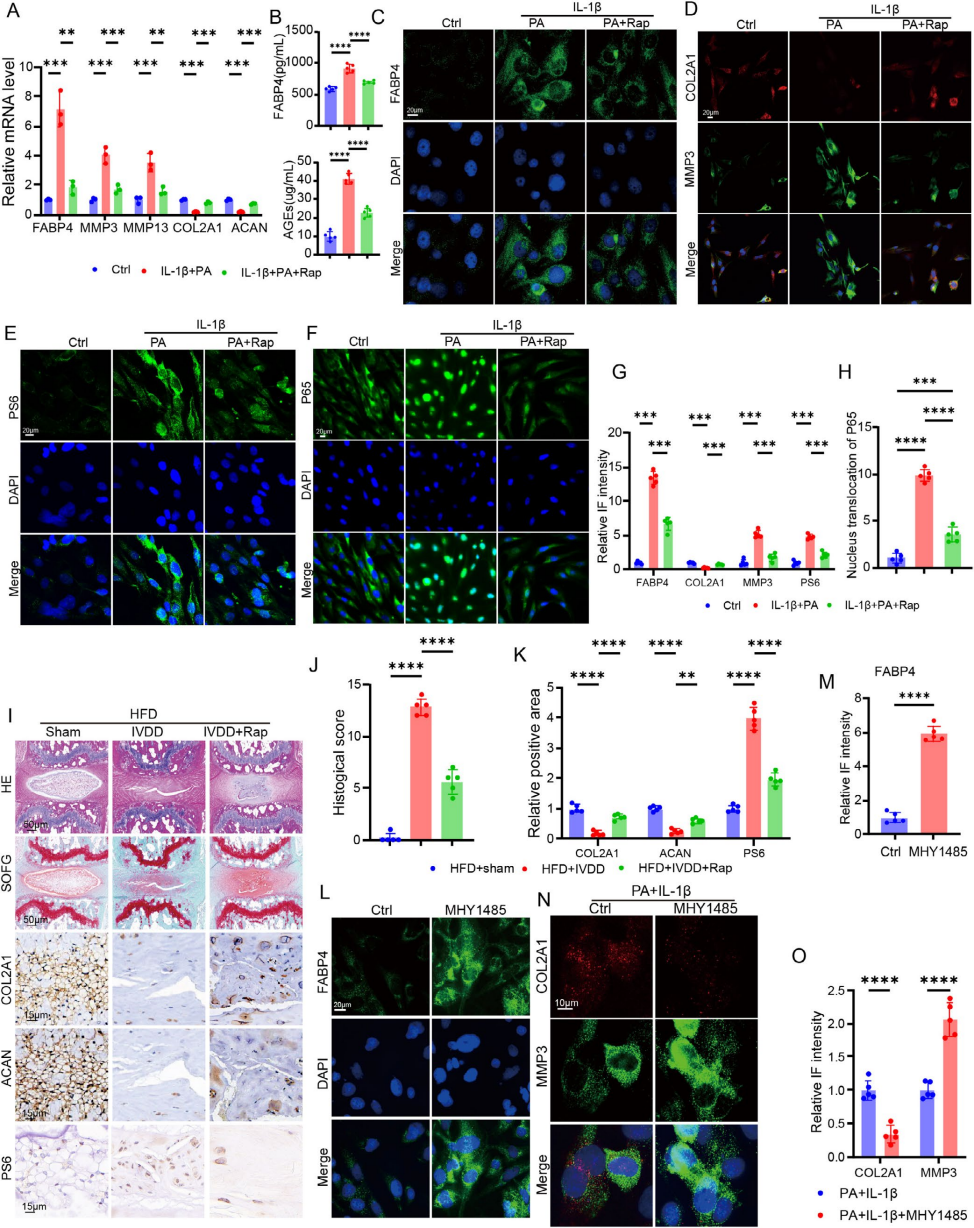
Obesity‐induced expression of FABP4 was regulated by the mTORC1 pathway. (A) RT‐qPCR for the expression of FABP4, ACAN, COL2A1, MMP3, and MMP13 in NPCs under lipotoxic condition treated with or without rapamycin (a specific inhibitor of the mTOR pathway) (*n* = 3). (B) ELISA result of FABP4 and AGEs levels under lipotoxic condition treated with or without rapamycin (*n* = 5). (C–F) IF staining for FABP4, COL2A1, MMP3, PS6, and p65 in NPCs under lipotoxic condition treated with or without rapamycin. (G) Quantitation results of the IF staining for FABP4, COL2A1, MMP3, and PS6 in NPCs under lipotoxic condition treated with or without rapamycin (*n* = 5). (H) Quantitation results of the IF staining for nucleus translocation of p65 in NPCs under lipotoxic condition treated with or without rapamycin (*n* = 5). (I) Degeneration and mTORC1 pathway activation evaluated by H&E, SOFG, and IHC staining of IVD tissue in sham, IVDD, and IVDD+ rapamycin groups, respectively. (J) Histological score of IVD tissue in sham, IVDD, and IVDD+ rapamycin groups, respectively (*n* = 5). (K) Quantitation results of the IHC staining for COL2A1, ACAN, and PS6 in sham, IVDD, and IVDD+ rapamycin groups, respectively (*n* = 5). (L and M) IF staining and quantitation results for FABP4 in NPCs treated with or without MHY1485 (an activator for mTOR pathway). N and O: IF staining and quantitation results for COL2A1 and MMP3 in NPCs treated with or without MHY1485 (an activator for mTOR pathway). *p < 0.05, **p < 0.01, ***p < 0.001, and ****p < 0.0001.

A recent study indicated that the activation of the mTORC1 signalling pathway could directly regulate FABP4 expression [28]. In addition, mTORC1 has been reported to be activated in an obesity state [29, 30]. Nevertheless, whether obesity‐induced expression of FABP4 was regulated by the mTORC1 pathway remains elusive. After the establishment of the obesity‐induced NPC model in vitro, we treated NPCs with or without rapamycin (a specific inhibitor of the mTOR pathway). The results of RT‐qPCR showed that PA and IL‐1β dramatically increased the expression of FABP4, MMP3, and MMP13, as well as the secretion of AGEs, but decreased the expression of ACAN and COL2A1 (Figure 4A). Nevertheless, blocking the mTOR signalling pathway using rapamycin (Rap) significantly ameliorated these deleterious effects (Figure 4A). In addition, inhibiting the activation of the mTORC1 pathway decreased the production of both FABP4 and AGEs induced by an obesity state in vitro (Figure 4B). IF staining further suggested that inhibiting the activation of the mTORC1 pathway significantly lowered the expression of FABP4 and MMP3 and enhanced the expression of COL2A1 (Figure 4C,D,G). Ribosomal protein S6 (PS6) is the critical downstream effector of mTORC1 [28]. Therefore, we evaluated the phosphorylation of S6, and the results showed that PA and IL‐1β enhanced the phosphorylation of S6, but the effect was mitigated by rapamycin (Figure 4E,G). In addition, we also explored the activation of NFκB signalling pathway and revealed that inhibiting the mTORC1 pathway lowered the nucleus translocation of p65 (Figure 4F,H).

Furthermore, we investigated the therapeutic effect of blocking the mTORC1 pathway on obesity‐related IVDD in vivo. H&E staining suggested that the administration of rapamycin obviously improved the IVD state in comparison to the IVDD group, including visible NP tissue and a relatively clear boundary between NP and AF tissue (Figure 4I,J). SOFG staining also demonstrated that blocking the mTORC1 pathway alleviated obesity‐induced IVDD in terms of the proteoglycan content (Figure 4I). Additionally, IHC staining confirmed the therapeutic effect of rapamycin on ECM homeostasis and the inactivation of the mTORC1 pathway (Figure 4I,K). We also evaluated the effects of activating mTORC1 using MHY1485 on FABP4 expression and ECM balance in NPCS. IF staining suggested that MHY1485 could increase the expression of FABP4 (Figure 4L,M). In addition, MHY1485 enhanced the damage of PA + IL‐1β to NPCs in vitro regarding ECM metabolism (Figure 4N,O).

Overall, these findings showed that the activation of the mTORC1 pathway stimulated FABP4, leading to obesity‐related IVDD.

### 3.5 FABP4 Promoted Angiogenesis to Exacerbate IVDD Under Lipotoxic Conditions

**FIGURE 5:**
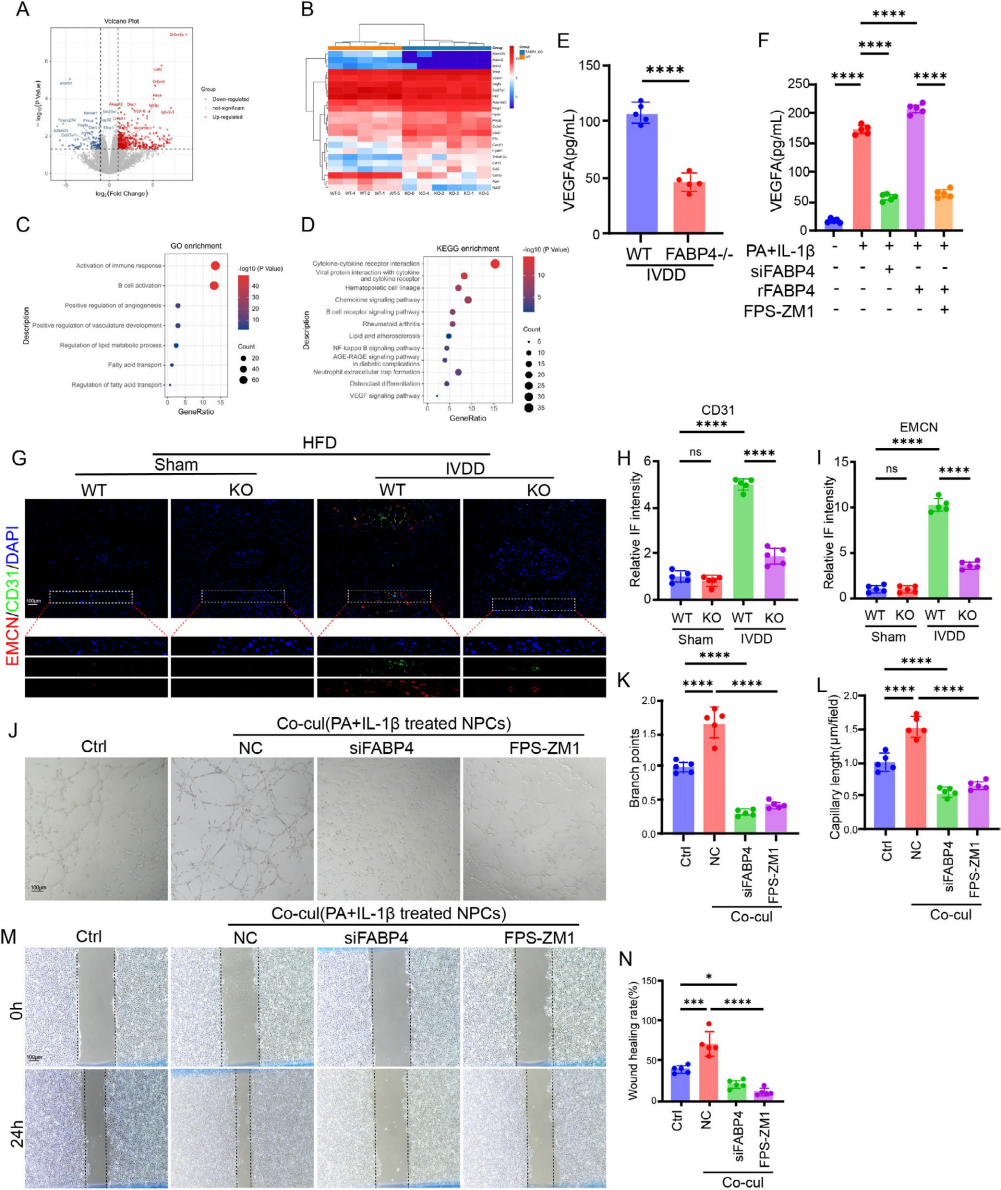
FABP4 promoted angiogenesis to exacerbate IVDD under lipotoxic condition. (A) Volcano plot of the DEGs of HFD‐induced IVDD from WT and KO mice. (B) Heart map of the DEGs of HFD‐induced IVDD from WT and KO mic (*n* = 5). (C) GO analysis of the DEGs of HFD‐induced IVDD from WT and KO mice. (D) KEGG analysis of the DEGs of HFD‐induced IVDD from WT and KO mice. (E) ELISA result of the VEGF level of the IVD tissue in WT and KO mice (*n* = 5). (F) ELISA result of the VEGF level of NPC degeneration model with or without FABP4 silencing or FPS‐ZM1 (*n* = 5). (G) IF staining for CD31 and EMCN in WT and KO mice from sham or IVDD groups under HFD condition, respectively. (H and I) Quantitation results of the IF staining for CD31 and EMCN in WT and KO mice from sham or IVDD groups under HFD condition, respectively (*n* = 5). (J) Tube formation assay of HUVECs in different groups. (K and L) Branch points and capillary length of HUVECs in different groups (*n* = 5). (M and N) Representative images and quantitation results of the scratch wound of HUVECs in different groups (*n* = 5). *p < 0.05, **p < 0.01, ***p < 0.001, and ****p < 0.0001.

Angiogenesis is another typically pathological characteristic of IVDD [31]. Next, we explored the role of FABP4 in angiogenesis. The results of bulk RNA sequencing suggested that rFABP4 could increase the expression of VEGF and angiogenesis (Figure 5A–D). We then performed the in vivo experiments and found that knocking out FABP4 decreased the production of VEGF in IVD tissue (Figure 5E). In vitro experiments suggested that silencing FABP4 lowered the production of VEGF in NPCs under lipotoxic conditions (Figure 5F). Notably, blocking the AGE‐RAGE pathway also lowered the increase in the secretion of VEGF induced by rFABP4 treatment (Figure 5F). These results demonstrated that FABP4 activated the angiogenesis‐related pathway via the AGEs‐RAGE signalling pathway. Then, we evaluated the ingrowth of new blood vessels into IVD tissue in WT and FABP4‐KO mice of obesity‐induced IVDD. IF staining suggested that there was no difference in the ratio of EMCN‐positive cells between WT and FABP4‐KO obese mice without needle surgery‐induced IVDD (Figure 5G–I). Nevertheless, compared to the WT obese mice without IVDD, the WT obese mice with IVDD showed a significant increase in the ratios of EMCN‐ or CD31‐positive cells, whereas knocking out FABP4 significantly alleviated this effect (Figure 5G–I).

Subsequently, we evaluated the effects of FABP4 on angiogenesis in vitro via co‐culturing degenerated NPCs with human umbilical vein endothelial cells (HUVECs). The NPC degeneration model was established using PA and IL‐1β to mimic inflammation‐related IVDD under obesity conditions. The results showed that co‐culture significantly promoted tube formation compared to the control group (Figure 5J–L). Nevertheless, either silencing FABP4 or blocking the AGEs signalling pathway using FPS‐ZM1 evidently alleviated co‐culture‐induced tube formation (Figure 5J–L). In addition, we evaluated the effects of FABP4 on HUVECs migration and found that HUVECs exposed to the NPC degeneration model displayed enhanced migration into the wounded area (Figure 5M,N). However, after inhibiting FABP4 or the AGEs signalling pathway, cell migration was reduced significantly (Figure 5M,N).

Collectively, obesity‐induced FABP4 could effectively promote angiogenesis, possibly via the AGEs‐RAGE signalling pathway.

### 3.6 Blocking the Interaction of VEGF and VEGFR2 Alleviated FABP4‐Induced Angiogenesis and IVDD

**FIGURE 6:**
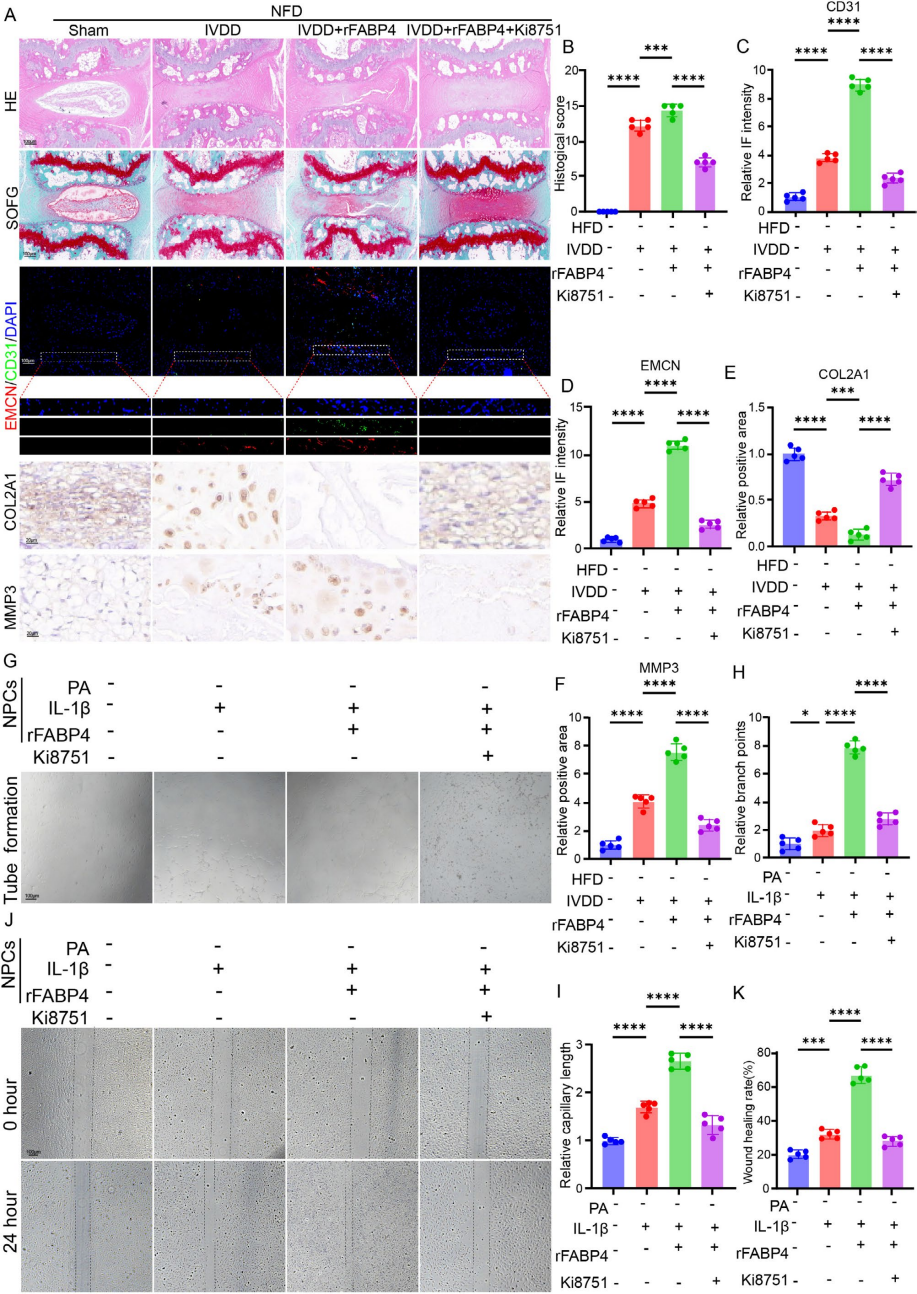
Blocking the interaction of VEGF and VEGFR2 alleviated FABP4‐induced angiogenesis and IVDD (A) Degeneration and angiogenesis evaluated by H&E, SOFG, and IHC staining of IVD tissue from sham, IVDD, IVDD+rFABP4, and IVDD+rFABP4 + Ki8751 groups, respectively. (B) Histological score of IVD tissue from sham, IVDD, IVDD+rFABP4, and IVDD+rFABP4 + Ki8751 groups, respectively (*n* = 5). (C and D): Quantitation results of IF staining for CD31 and EMCN of IVD tissue from sham, IVDD, IVDD+rFABP4, and IVDD+rFABP4 + Ki8751 groups, respectively (*n* = 5). (E and F) Quantitation results of IHC staining for COL2A1 and MMP3 of IVD tissue from sham, IVDD, IVDD+rFABP4, and IVDD+rFABP4 + Ki8751 groups, respectively (*n* = 5). (G) Tube formation assay of HUVECs in different groups. (H and I) Branch points and capillary length of HUVECs in different groups (*n* = 5). (J and K) Representative images and quantitation results of the scratch wound of HUVECs in different groups (*n* = 5). *p < 0.05, **p < 0.01, ***p < 0.001, and ****p < 0.0001.

Given that abnormal angiogenesis can accelerate the progression of IVDD, we evaluated the therapeutic effects of blocking VEGF signalling on IVDD. H&E and SOFG staining showed that under a normal diet, the administration of rFABP4 deteriorated needle‐induced IVDD, whereas blocking VEGF signalling using Ki8751 obviously ameliorated the degenerated condition, as shown by the morphology and cellularity of NP tissue and proteoglycan content within IVD tissue (Figure 6A,B). Regarding angiogenesis, IF staining suggested that the administration of rFABP4 enhanced needle‐induced expression of angiogenesis‐related markers, CD31 and EMCN, whereas blocking VEGF signalling using Ki8751 obviously ameliorated the pro‐angiogenesis effects (Figure 6A,C,D). IHC staining for COL2A1 and MMP3 further confirmed the therapeutic effects of blocking VEGF signalling on IVDD (Figure 6A,E,F).

Further, we evaluated the therapeutic effects of blocking VEGF signalling on angiogenesis via co‐culturing the NPC degeneration model with HUVECs. The NPC degeneration model was established using PA and IL‐1β to mimic obesity‐related IVDD. The tube‐forming assay showed that co‐culturing with the NPC degeneration model strengthened the ability of tube formation of HUVECs and the administration of rFABP4 further enhanced this effect (Figure 6G–I). However, blocking the VEGF signalling pathway significantly alleviated the pro‐angiogenic effects (Figure 6G–I). In addition, we evaluated cell migration. Similarly, we found that blocking the VEGF signalling pathway also significantly inhibited the migration of HUVECs into the wounded area (Figure 6J,K).

Therefore, blocking the interaction of VEGF and VEGFR2 alleviated FABP4‐induced angiogenesis and IVDD.

### 3.7 Inhibition of FABP4 Alleviated Obesity‐Related IVDD via Restoring ECM Balance and Inhibiting Angiogenesis

**FIGURE 7:**
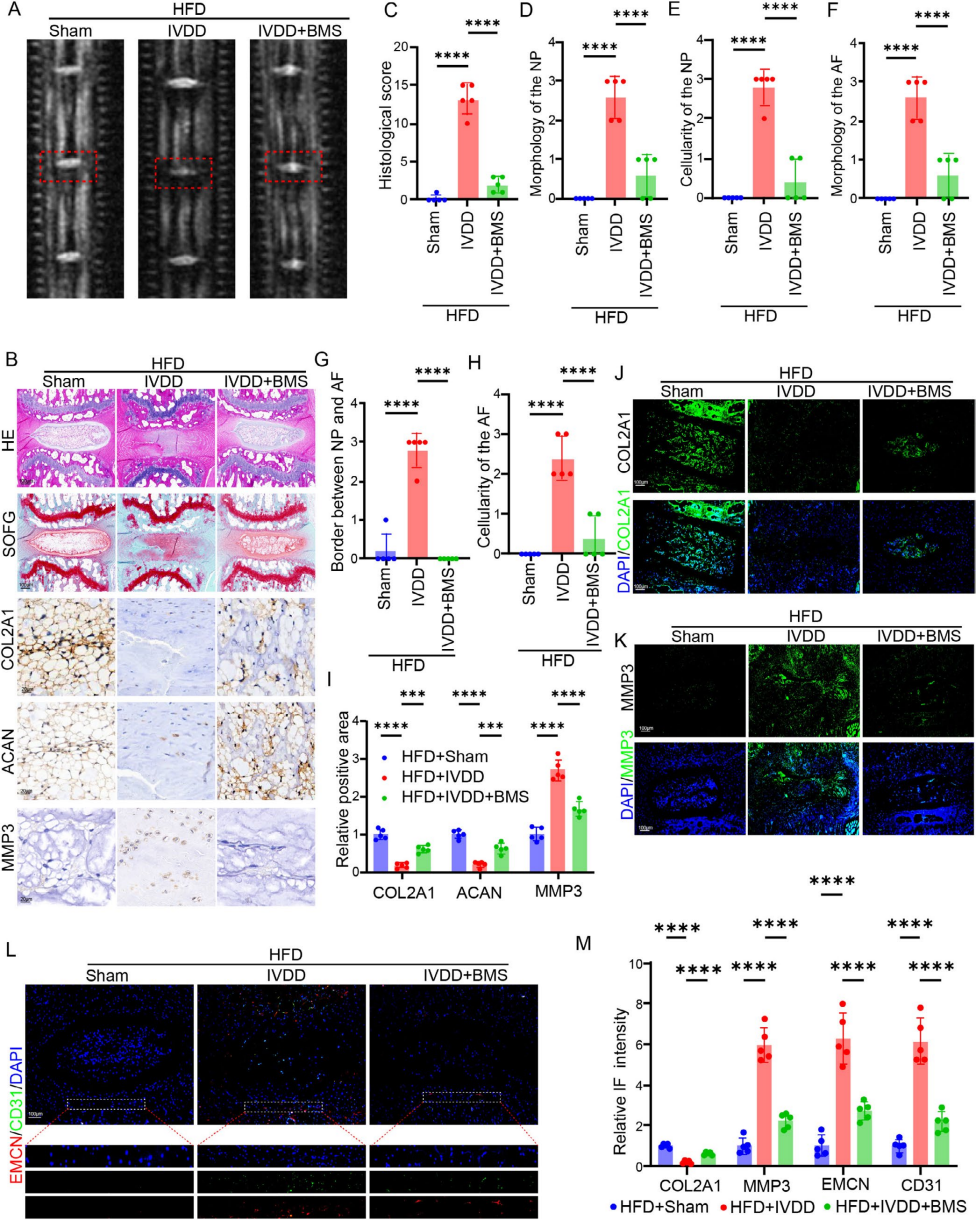
Inhibition of FABP4 alleviated obesity‐related IVDD via restoring ECM balance and inhibiting angiogenesis (A) Representative MRI images of IVD in sham, IVDD, and IVDD+BMS groups, respectively. (B) Degeneration evaluated by H&E, SOFG, and IHC staining of IVD tissue from sham, IVDD, and IVDD+BMS groups, respectively. (C–H) Histological score of IVD from sham, IVDD, and IVDD+BMS groups, respectively (*n* = 5). (I) Quantitation results of IHC staining for COL2A1, ACAN, and MMP3 of IVD from sham, IVDD, and IVDD+BMS groups, respectively (*n* = 5). (J–L) IF staining for COL2A1, MMP3, CD31, and EMCN of IVD from sham, IVDD, and IVDD+BMS groups, respectively. (M) Quantitation results of IF staining for COL2A1, MMP3, CD31, and EMCN of IVD from sham, IVDD, and IVDD+BMS groups, respectively (*n* = 5).

Given that FABP4 was the critical mediator of NPC dysfunction and IVDD, BMS309403 (a FABP4 inhibitor, MedChemExpress, Shanghai, China) was used in the IVDD model under HFD conditions to evaluate the therapeutic effects. Firstly, we established an obesity mouse model via a three‐month HFD. Then, a needle‐induced IVDD model was established. For the treatment group, BMS309403 was given to the mice via intradiscal injection (5 mg/kg) once a week for the first two weeks until the end of one month after surgery. We firstly evaluated the degeneration of mouse IVDs in each group using T2‐weighted MRI. The results showed that in obese mice, the needle obviously resulted in the loss of water signal within the IVD, whereas inhibiting FABP4 using BMS309403 significantly alleviated the water signal loss (Figure 7A). Further, we evaluated the histological changes of the IVD. H&E staining suggested that compared to the sham group, the volume of NP tissue was obviously decreased and the boundary between NP and AF tissue was not clear in the HFD + IVDD group (Figure 7B). Nevertheless, treatment with BMS309403 significantly alleviated the damage to NP tissue resulting from the needle, accompanied by a decreased degeneration score (Figure 7B–H). We further evaluated the proteoglycan content within the IVD tissue using SOFG staining, and the results showed that many NPCs with large vacuoles and more proteoglycan content existed in the gelatinous structure of NP tissues from the sham group, whereas the needle markedly resulted in the disappearance of vacuoles and reduced proteoglycans in NPCs in the two IVDD groups (Figure 7B). However, mice in the treatment (IVDD+BMS) group showed significantly alleviated decreases in the proteoglycan content (Figure 7B). ECM homeostasis was evaluated via IHC staining, and the results showed that the needle resulted in ECM degradation, including decreased expression of ACAN and COL2A1, and increased expression of MMP3 (Figure 7B,I). However, treatment with BMS309403 significantly rescued these effects (Figure 7B,I). IF staining for COL2A1 and MMP3 of IVD tissue also showed a consistent tendency to that of IHC staining (Figure 7J,K,M). We also assessed the state of angiogenesis via IF staining for EMCN and CD31. The results demonstrated that the needle increased the ratio of EMCN‐ and CD31‐positive cells within the IVD tissue, but treatment with BMS309403 evidently decreased the ratio in the IVDD+BMS group (Figure 7L,M).

**FIGURE 8:**
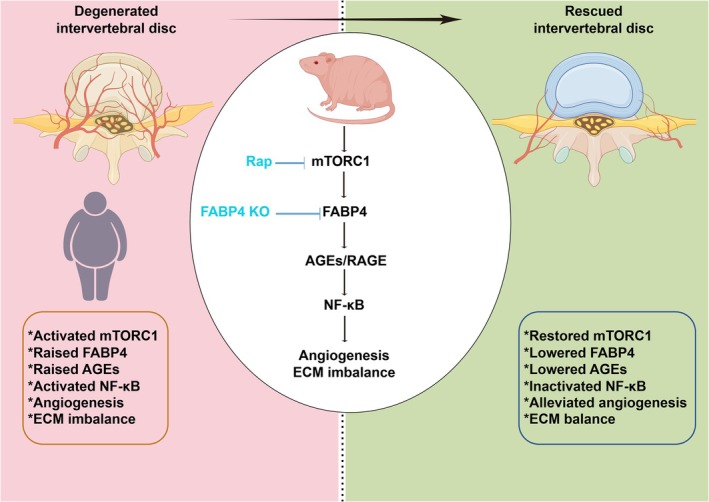
A diagram of the molecular mechanism in this study.

Therefore, these data indicate that BMS309403 targeting FABP4 can effectively ameliorate nucleus pulposus dysfunction and angiogenesis in obesity‐related intervertebral disc degeneration. A diagram of the molecular mechanism is presented in Figure 8.

## 4 Discussion

As society's economy develops, the rate of obesity is rising, with over 1.9 billion people globally being overweight [24]. Obesity refers to an excessive buildup of fat in the body that poses a threat to human health. The correlation between obesity and IVDD has been previously demonstrated, and adipokines play a critical role in regulating homeostasis within IVDD [3, 6, 7]. Early study found that the risk of IVDD was 1.3–1.8‐fold higher in overweighed individuals than those without obesity [32]. A meta‐analysis study further uncovered the relationship between these two diseases [33]. However, the majority of these studies are cohort or cross‐sectional in nature and cannot establish a definitive causal link. Additionally, several studies have presented conflicting evidence that contradicts the proposed association [34]. T The link between obesity and IVDD requires more research. In this study, we initially uncovered the causal relationship between obesity and IVDD using a two‐sample Mendelian randomization approach. Moreover, experiments conducted on live animals confirmed that obesity enhances the speed and severity of IVDD progression. This study's findings suggest that weight loss should be prioritised in obese populations to lower the risk of IVDD and LBP.

Given that the critical role of FABPs in lipid transport, we firstly evaluated the expression of FABPs in NPCs both in vitro and in vivo during the obesity state. The use of PA in our cell model was critical to recapitulating the lipotoxic environment associated with obesity. PA is a major component of dietary fats and is released in large quantities during the lipolysis of adipose tissue in obese individuals [35]. Elevated PA levels have been shown to induce endoplasmic reticulum (ER) stress, mitochondrial dysfunction, and inflammation, all of which are hallmarks of obesity‐related metabolic disorders [36]. By treating NPCs with PA, we were able to mimic the lipotoxic effects of obesity and investigate the role of FABP4 in mediating these effects. This approach allowed us to establish a direct link between obesity‐induced lipotoxicity and IVDD pathogenesis, providing valuable insights into the molecular mechanisms underlying this relationship. In this study, RT‐qPCR and histological staining revealed that FABP4 was among the most upregulated FABPs in degenerated human NP tissue or human primary NPCs due to obesity. FABP4, which is also referred to as A‐FABP or aP2, is a newly identified adipokine that functions as a fatty acid chaperone, linking intracellular lipids to biological targets and signalling pathways [37]. FABP4 has been linked to various components of metabolic syndrome in mice, including type 2 diabetes and atherosclerosis, and blocking FABP4 improved metabolism‐related issues [38, 39]. In recent years, FABP4 was reported to participate in regulating orthopaedic‐related diseases. Chen et al. found that the expression of FABP4 correlated positively with the severity of rheumatic arthritis [40]. Yang et al. reported that inhibiting FABP4 activity showed anti‐osteoarthritis properties and protected against cartilage degradation in OA patients [41]. Nevertheless, How FABP4 was regulated during obesity‐related IVDD and whether it plays a critical role during the progression of IVDD remains elusive. In this present study, we firstly revealed that either overexpression of FABP4 or exogenous administration of rFABP4 resulted in ECM imbalance and NPC degeneration. However, knocking out FABP4 in mice significantly alleviated the progression of IVDD in the obesity state via regulating ECM homeostasis. Collectively, these findings indicated that FABP4 is a potential therapeutical target for the treatment of IVDD.

To further investigate the intrinsic mechanism of FABP4 in regulating IVDD, we performed the bulk RNA sequencing of human primary NPCs treated by rFABP4. The function analysis suggested that FABP4 dramatically activated AGEs and NF‐κB signalling pathways. AGEs are the final products formed when proteins, lipids, and nucleic acids undergo non‐enzymatic glycosylation with glucose and other reducing sugars [42]. Previous study reported that the level of AGEs was correlated with the severity of atherosclerosis [43]. In a recent study, Tseng et al. revealed that AGEs could promote IVDD via activating MMPs [44]. Likewise, Luo et al. found that AGEs could contribute to NPC apoptosis and IVDD via disturbing Ca^2+^ homeostasis and endoplasmic reticulum (ER) stress [45]. However, inhibiting the production of AGEs protected IVD against degeneration [46]. In this study, we were the first to show that FABP4 enhances the production of AGEs in NPCs in a dose‐dependent way. In addition, we have clarified that blocking the AGEs‐RAGE signalling pathway ameliorated FABP4 accumulation‐mediated ECM disequilibrium both in vivo and in vitro. OF note, we also showed that FABP4 could promote the activation of NFκB signalling pathway. Our previous study reported that NFκB signalling pathway was a critical pathway during the initiation and development of IVDD [47, 48]. In fact, NFκB signalling pathway widely participated in the regulation of ECM degradation, inflammation, and oxidative stress in degenerated IVD tissue [49]. In addition, blocking the activation of NFκB signalling pathway using JSH23 evidently alleviated either rFABP4‐ or AGEs‐mediated NPC degeneration. Collectively, this present study demonstrated a new regulation axis, FABP4/AGEs‐RAGE signalling axis to activate NFκB signalling pathway, which will facilitate a better understanding of the pathological mechanism between obesity and IVDD.

It has been well established that the Mammalian target of rapamycin C1 (mTORC1) pathway can regulate the progression of IVDD. According to Ito et al., selectively targeting mTORC1/RAPTOR can prevent inflammation‐induced apoptosis, senescence, and ECM breakdown [50]. Another two studies from Kakiuchi et al. and Yurube et al. revealed that pharmacological inhibition of mTORC1 protected against inflammation‐induced IVDD [51, 52]. Indeed, the mTORC1 signalling pathway combines various bio‐environmental signals, including growth factors and nutritional conditions, to regulate the growth of eukaryotic cells [53]. In a recent study, Li et al. found that a high fat diet‐induced obesity could activate the mTORC1 pathway and lead to depressive behaviours [54]. It is still unclear if the activation of the mTORC1 pathway played a role in increasing FABP4 transcription. In this present study, our findings both in vivo and in vitro demonstrated that obesity could result in the increased phosphorylation of S6 (ribosomal protein S6 (S6)), a critical downstream effector of mTORC1. Further, we found that activating mTOR increased the expression of FABP4, whereas inhibiting the activation of the mTORC1/PS6 axis decreased the expression of FABP4. In addition, inhibiting the mTORC1/PS6 axis using rapamycin showed significantly protective effects on obesity‐induced NPC degeneration. The regulation of FABP4 by mTORC1 was also observed in rheumatoid arthritis [28]. Therefore, the upregulation of FABP4 within degenerated NPCs in the obesity state was controlled by the activation of the mTORC1/PS6 axis cascade.

Angiogenesis is another typically pathological characteristic of IVDD. Under normal circumstance, IVD tissue is an avascular structure except for the outer AF tissue, and healthy NPCs express various angiogenesis inhibitors, such as seme3A and chondromodulin [55]. Previous studies demonstrated the close relationship between angiogenesis and inflammation due to infiltration of immune cells into IVD tissue [1, 31, 56]. In addition, the ingrowth of blood vessels into IVD tissue can also trigger innervation, leading discogenic pain [57]. Our findings provided experimental evidence that FABP4 increased the expression of VEGF, a key factor in regulating angiogenesis [58]. In vivo experiments suggested that obesity resulted in the increase of the ratio of Emcn‐positive cells within IVD tissue, whereas knocking out FABP4 ameliorated this pathological alternation. In vitro experiments demonstrated the administration of FABP4 contributed to angiogenesis. Importantly, inhibiting VEGF‐VEGFR signalling pathway alleviated recombinant FABP4‐mediated angiogenesis. Therefore, these findings indicated that in addition to activating AGEs‐RAGE/NFκB signalling pathway, FABP4‐induced angiogenesis would further exacerbate IVDD progression.

In addition to activating the AGEs‐RAGE/NF‐κB pathway, FABP4 may also engage with other intersecting signalling cascades that exacerbate IVDD pathogenesis. FABP4 has been implicated in promoting oxidative stress, which can lead to mitochondrial dysfunction and ROS accumulation and can further activate NF‐κB, thereby amplifying inflammatory responses within the NP tissue [59]. Beyond the canonical NF‐κB activation, AGEs‐RAGE signalling is also known to interact with the PI3K/Akt pathway, which regulates cellular survival, inflammation, oxidative stress, and autophagy [60]. Interestingly, our RNA‐seq analysis revealed enrichment of PI3K/Akt‐related genes in FABP4‐overexpressing NPCs, suggesting that this pathway might contribute to IVDD progression by mediating ECM catabolism and angiogenesis. By broadening our analysis beyond the AGEs‐RAGE/NF‐κB pathway, we now propose that mTORC1 activation, oxidative stress, ER stress, and PI3K/Akt signalling may serve as additional mediators of FABP4‐induced IVDD pathology. Future studies targeting these pathways in combination with FABP4 inhibition might also be possible therapeutic strategies for IVDD treatment in obesity‐related conditions.

Based on the results above, suppressing FABP4 can be an alternative for the treatment of IVDD. BMS309403 is a newly identified FABP4 inhibitor. Therefore, we performed the pre‐clinical experiments. Our findings showed that BMS309403 treatment could effectively mitigate needle‐induced IVDD in an obese state by inhibiting angiogenesis, which was consistent with the results of a previous study that BMS309403 could repress blood vessel‐formation‐related behaviour of HUVEC [28, 61]. In addition, we evaluated the ECM balance after treatment and found that the administration of BMS309403 dramatically restored the ECM balance in needle‐induced IVDD in obese mice. Overall, the results mentioned above highlight the potential clinical benefits of using a FABP4 inhibitor for treating IVDD.

Despite the critical role of FABP4 in obesity‐related IVDD, there are still several limitations that should be acknowledged. Firstly, this present study showed that FABP4 was a critical factor to induce IVDD in an obesity state, and inhibiting FABP4 using BMS309403 showed promising therapeutic effects. However, the optimal dose and treatment duration remain to be determined, and the side effects of long‐term administration are still unclear. Therefore, further studies are needed to evaluate the potential metabolic side effects and chronic effects of FABP4 inhibition, particularly in obese individuals who may already have compromised metabolic health. Secondly, in our study, we used a single dose of BMS309403 and PA based on previous literature. However, the optimal dosing regimen for FABP4 inhibitors in the context of IVDD has not been established. Variations in drug metabolism, bioavailability, and tissue‐specific effects may influence the efficacy and safety of FABP4 inhibitors. Therefore, dose‐ranging studies are necessary to determine the most effective and safe dosage for therapeutic applications. In our future research, we will further explore the dose optimisation of the FABP4 inhibitor, as well as the potential side effects, which we have added in the section on limitations. Thirdly, although we proved that FABP4 could increase the production of AGEs, the intrinsic mechanism between FABP4 and AGEs remains elusive. Further studies are needed to completely elucidate the regulatory mechanism of FABP4 in AGEs. Finally, this study focused on NPC‐derived FABP4 and IVDD. However, most previous studies also showed that FABP4 was also secreted by macrophages and adipocytes. Therefore, further study with gene conditionally knocked‐out mice (NPC‐specific, macrophage‐specific, and adipocyte‐specific mice) investigating the crosstalk between NPCs and macrophages or adipocytes is also required.

In conclusion, this present study demonstrated that obesity is the major risk factor for the development of IVDD, and that lipid transport‐related cytokine, FABP4, plays the critical role in regulating ECM homeostasis in NPCs and angiogenesis via the AGEs‐RAGE/NFκB signalling pathway and VEGF/VEGFR axis, respectively. Finally, genetically modulating or pharmacologically inhibiting the expression of FABP4 showed effective therapeutic potential to alleviate IVDD via remodelling ECM balance and anti‐angiogenesis. Collectively, our results provide a potential therapeutic target, FABP4, for alleviating NPC dysfunction and ECM catabolism associated with obesity‐induced IVDD.

## Author Contributions

Investigation and the original manuscript writing: Lin Han, Fudong Li, Huiqiao Wu, and Weiheng Wang; Data collection: Peiwen Chen; Software: Weicheng Xia; Design, funding, and manuscript reviewing: Yang Liu, Kaiqiang Sun, Wenbo Lin.

## Ethics Statement

This present study was approved by the Animal Research Ethics Committee at our institution.

## Conflicts of Interest

The authors declare no conflicts of interest.

## Supporting information


**Figure S1:**The expression levels of FABPs. (A) The mRNA levels of FABP1, FABP2, FABP3, FABP5, FABP6, FABP7, and FABP12. (B) The level of FABP4 in the serum of mice treated with NFD or HFD.

## Data Availability

The data that support the findings of this study are available from the corresponding author upon reasonable request.
