# Supplementary figures and images for: Targeting FABP4 to Inhibit AGEs‐RAGE/NF‐κB Signalling Effectively Ameliorates Nucleus Pulposus Dysfunction and Angiogenesis in Obesity‐Related Intervertebral Disc Degeneration
Source: Cell Prolif. 2025 Mar 16;58(9):e70021. doi: 10.1111/cpr.70021 (PMC12414640; doi:10.1111/cpr.70021)

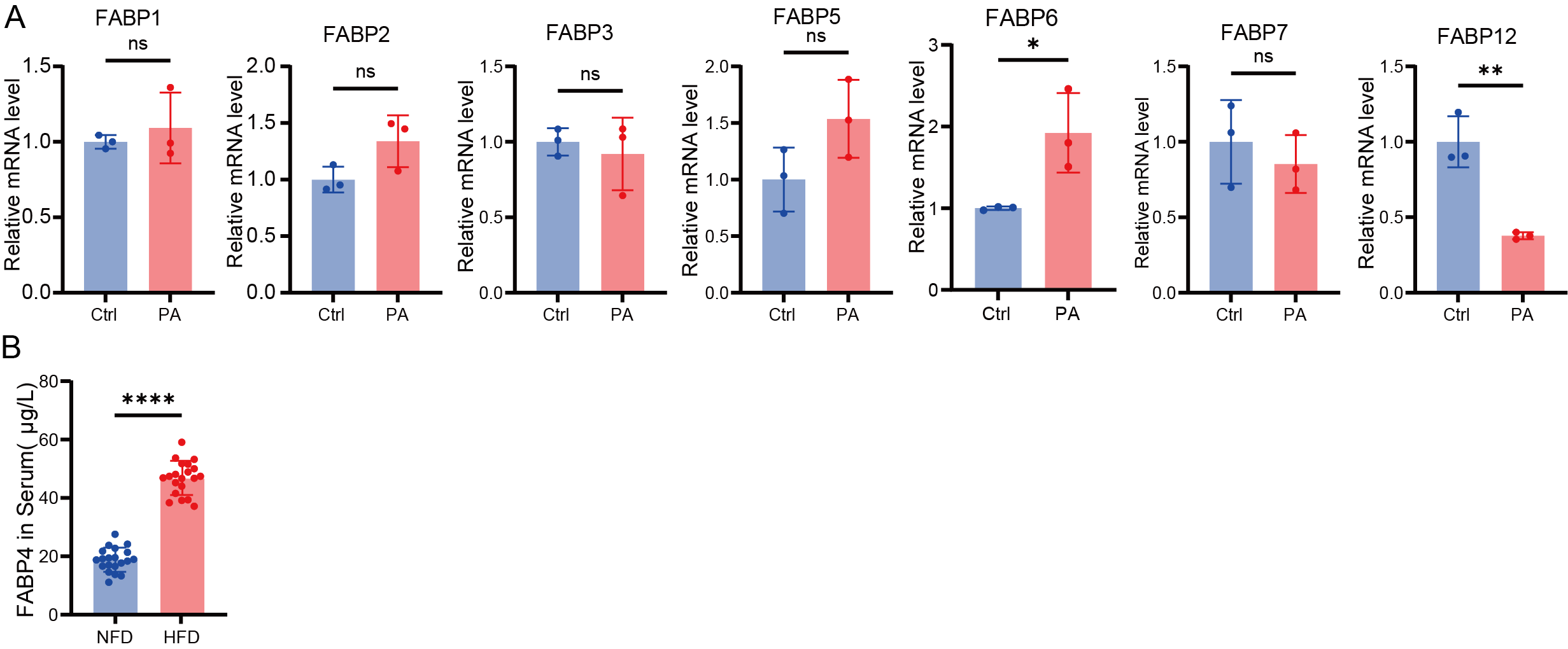

Supplement: Supplementary file 1 — Figure S1:The expression levels of FABPs. (A) The mRNA levels of FABP1, FABP2, FABP3, FABP5, FABP6, FABP7, and FABP12. (B) The level of FABP4 in the serum of mice treated with NFD or HFD. [file CPR-58-e70021-s001.tif]
